# Universal Germline-Genetic Testing for Breast Cancer: Implementation in a Rural Practice and Impact on Shared Decision-Making

**DOI:** 10.1245/s10434-023-14394-3

**Published:** 2023-10-09

**Authors:** Charles Shelton, Antonio Ruiz, Lauren Shelton, Hannah Montgomery, Karen Freas, Rachel E. Ellsworth, Sarah Poll, Daniel Pineda-Alvarez, Brandie Heald, Edward D. Esplin, Sarah M. Nielsen

**Affiliations:** 1 The Outer Banks Hospital, Nags Head, NC USA; 2 Carolina Surgical Care, Chesapeake, VA USA; 3 Invitae Corporation, San Francisco, CA USA

## Abstract

**Background:**

Whereas the National Comprehensive Cancer Network (NCCN) criteria restrict germline-genetic testing (GGT) to a subset of breast cancer (BC) patients, the American Society of Breast Surgeons recommends universal GGT. Although the yield of pathogenic germline variants (PGV) in unselected BC patients has been studied, the practicality and utility of incorporating universal GGT into routine cancer care in community and rural settings is understudied. This study reports real-world implementation of universal GGT for patients with breast cancer and genetics-informed, treatment decision-making in a rural, community practice with limited resources.

**Methods:**

From 2019 to 2022, all patients with breast cancer at a small, rural hospital were offered GGT, using a genetics-extender model. Statistical analyses included Fisher’s exact test, *t*-tests, and calculation of odds ratios. Significance was set at *p* < 0.05.

**Results:**

Of 210 patients with breast cancer who were offered GGT, 192 (91.4%) underwent testing with 104 (54.2%) in-criteria (IC) and 88 (45.8%) out-of-criteria (OOC) with NCCN guidelines. Pathogenic germline variants were identified in 25 patients (13.0%), with PGV frequencies of 15 of 104 (14.4%) in IC and ten of 88 (11.4%) in OOC patients (*p* = 0.495). GGT informed treatment for 129 of 185 (69.7%) patients.

**Conclusions:**

Universal GGT was successfully implemented in a rural, community practice with > 90% uptake. Treatment was enhanced or de-escalated in those with and without clinically actionable PGVs, respectively. Universal GGT for patients with breast cancer is feasible within rural populations, enabling optimization of clinical care to patients’ genetic profile, and may reduce unnecessary healthcare, resource utilization.

**Supplementary Information:**

The online version contains supplementary material available at 10.1245/s10434-023-14394-3.

Breast cancer (BC) care and outcomes differ for women living in rural compared with metropolitan areas, with lower incidence but higher mortality.^1^ One contributor to less favorable outcomes for women in rural communities is access to care. For example, only 44% and 30% of hospitals in Upstate New York offered radiation oncology and breast surgery services, respectively.^2^ This limited access to comprehensive breast care results in individuals within rural communities required to travel longer distances for treatment, which results in increased economic costs, anxiety, and time away from home and family.^3^ These barriers may influence treatment choice. For example, women living in rural areas are less likely to undergo breast-conserving therapy (BCT) because of decreased access to radiotherapy (RT) facilities and to have delayed initiation of primary therapy.^4,5^ Guideline-discordant treatment has been associated with decreased BC-specific and overall survival.^6^

Germline-genetic testing (GGT) may influence treatment choices, because surgical, RT, chemotherapy, precision therapy, and surveillance strategies have been established for individuals with pathogenic germline variants (PGVs) in several cancer-predisposition genes.^7–9^ For example, BC patients with PGVs in *BRCA1* and *BRCA2* are at increased risk for developing contralateral breast cancer compared with noncarriers.^10–12^ Whereas contralateral prophylactic mastectomy (CPM) has been shown to reduce risk of contralateral cancer by 93%,^13^ evidence about whether CPM improves BC survival in *BRCA* carriers is conflicting.^14^ The American Society of Clinical Oncology, American Society for Radiation Oncology, and Society of Surgical Oncology recommend that presence of a PGV in *BRCA1/2* does not preclude BCT and that BCT, ipsilateral therapeutic mastectomy and CPM should be discussed.^15^ National Comprehensive Cancer Network (NCCN) guidelines recommend discussing the option of risk-reducing bilateral mastectomy (BLM) in patients with PGVs in *BRCA1*, *BRCA2*, and *PALB2*.^16^ In contrast, there is currently insufficient evidence for the benefit of BLM with other PGVs. Beyond surgery, identification of patients with PGVs may lead to changes in medical and radiation oncology treatments that include the use of poly (ADP-ribose) polymerase (PARP) inhibitors for *BRCA1/2-*positive patients with high-risk, early-stage or metastatic disease, platinum-based chemotherapy for *BRCA1/2-*associated, metastatic, triple-negative disease, avoidance of RT in those with PGVs in *TP53*, and accelerated, partial, breast irradiation (APBI) in patients without PGVs in *BRCA1/2*.^15–19^

Despite the potential contribution of GGT to treatment choice in patients with BC, not all societal guidelines endorse universal GGT. Rather, guidelines, such as those issued by NCCN restrict GGT to those with the highest likelihood of having a PGV in a cancer-predisposition gene.^16^ While these guidelines were originally developed to identify women at high-risk for having a PGV in *BRCA1/2*, current criteria are ineffective to identify women with non-BRCA PGVs.^20^ In addition, several studies found that approximately one-half of all women with a PGV associated with altered management strategies were not eligible for GGT under current guidelines.^21–24^ In response to these studies, the American Society of Breast Surgeons (ASBrS) issued consensus guidelines that universal GGT should be available to all women with a current or previous diagnosis of BC.^25^

Despite the clear implications of GGT in cancer care, the implementation, uptake, and clinical utility of universal GGT in rural community practices has not been studied. Barriers to care may affect the willingness or ability of rural patients to pursue GGT.

The Outer Banks Hospital (TOBH) is a rural, critical-access hospital located within the eastern, coastal region of North Carolina. It is a joint venture to increase access to rural inhabitants by Chesapeake Regional Hospital and East Carolina University Health. A 2014 community analysis found that BC mortality was 19% higher for residents of Eastern North Carolina compared with the rest of the state.^6^ A retrospective analysis of risk factors in 165 BC patients diagnosed at TOBH found that 33% of patients had at least one first-degree relative with BC and 55% met NCCN GGT guidelines,^26^ which is in line with a large study of Utah residents that found 63% of BC patients met NCCN GGT guidelines, with 54% attributable to family history.^27^

Universal GGT in this population may benefit both patient outcomes and curtail treatment costs, with more aggressive approaches tailored to patients with PGVs and de-escalation of unnecessary interventions in those without. In 2019, in response to the ASBrS consensus guideline, TOBH integrated universal testing for all patients diagnosed with BC into prospective-care pathways to measure the impact of GGT on outcomes. This study evaluates the implementation of universal testing in a rural community oncology practice, including the frequency of PGVs in patients who were in-criteria (IC) and out-of-criteria (OOC) with respect to NCCN GGT guidelines, as well as the GGT-based changes to treatment and clinical management.

## Methods and Materials

### Patient Population

Beginning in 2018, TOBH collaborated with a breast surgeon at a large, community hospital in Chesapeake, VA, to expand breast-specific, clinical resources available to its rural population. Starting in 2019, all patients who had pathology-confirmed BC diagnosed at TOBH were eligible for GGT, consistent with ASBrS guidelines.

All locally diagnosed patients were prospectively presented to a multidisciplinary, breast-tumor board if any component of their care was to be provided at TOBH. The tumor boards met biweekly to discuss treatment plans for all patients newly diagnosed with primary or recurrent BC. These tumor boards were staffed by breast surgeons, pathologists, nurse navigators, radiation and medical oncologists, genetics-trained nurses, and radiologists. Nurse genetic extenders (NGE) were trained through the City of Hope’s Intensive Course in Genetic Cancer Risk Assessment, which is one of the various qualifications cited by the National Accreditation Program for Breast Centers to perform GGT and counseling.^28–30^ Additionally, two of our oncology providers were certified through additional genetics training. Providers discussed GGT results, clinical stage, imaging, pathology, and treatment options prospectively with all patients and recommended clinical-management strategies.

### Pre- and Post-test Genetic Education

This project was approved in July 2019 through an institutional review board at East Carolina University (UMCIRB 19-001052). During the pretest education session, NGEs obtained written, informed consent from patients for GGT. The majority of testing was performed at time of tissue confirmation of malignancy by the NGE embedded in the surgical clinic. All patients had private or government insurance and were responsible for additional costs associated with GGT. Providers charged one facility fee, which included genetic-counseling services. The fees for GGT were billed by the laboratories that performed the testing.

### Genetic Testing

NGEs collected and submitted saliva specimens to commercial laboratories for GGT. Multigene panel testing (median 47 genes) was performed for the majority of patients. Patients were grouped as PGV-positive if ≥ 1 PGV was detected, variant of uncertain significance (VUS) if ≥ 1 VUS and no PGVs were detected, or negative if no PGVs or VUS were detected. Genes with PGVs were grouped according to overall (absolute) risk of any cancer (Table S1).^31,32^

In-person disclosure of GGT results and post-test counseling were performed by the NGE/provider before patients were scheduled for primary therapy. All patients with PGVs were offered additional counseling with certified, genetic counselors employed by testing laboratories.

### Data Collection and Analysis

Demographic, clinical, and treatment data, including GGT results, were extracted from the patients’ EHR. Questionnaires administered to patients solicited information regarding family history of cancer and/or GGT per standard workflow. IC or OOC status was determined by using the NCCN criteria from the year of diagnosis. Preferred GGT-guided, clinical-management strategies from each treatment provider (surgeon, medical and radiation oncologist, NGE) were recorded at the time of the tumor board. Patient-reported outcomes were additionally collected by the research coordinator less than 6 months after treatment to measure the patients’ perspectives on GGT impact. Study data were collected and managed by using REDCap electronic data capture tools hosted at Invitae Corporation.^33,34^

Evaluation of compliance with NCCN management recommendations was performed by using the patient inclusion/exclusion criteria described by Kurian et al.^35^ Briefly, to determine whether BLM was overused, all patients diagnosed with unilateral, stage 0–III BC were included. For analysis of underuse of RT, patients aged ≥ 70 years who were diagnosed with stage I, hormone receptor-positive, *ERBB2*-negative tumors were excluded. All other patients with stage 0–III BC treated with BCT were included. Odds ratios, *t*-tests, and Fisher’s exact tests were performed. A value of *p* < 0.05 was used to define significance, and Bonferroni multiple testing correction was applied where appropriate.

## Results

Of 210 patients diagnosed with BC from 2019 to 2022 at TOBH, 192 (91.4%) underwent GGT and received results. Reasons for not undergoing GGT or not receiving results included: sample failure and no subsequent sample sent (*n* = 2); patient declined testing due to age (> 80 years, *n* = 3); insurance denied coverage (*n* = 2); or patient evaluated by a community surgeon who was not aware of the ASBrS guidelines (*n* = 11). Within the tested group, 104 (54.2%) were IC at the time of GGT, and 88 (45.8%) were OOC. Two patients who were OOC at the time of testing in 2020 would have been IC using the NCCN 2022 guidelines; neither patient had a PGV. IC patients (mean age, 58.6 [range 32–91] years) were significantly younger than OOC patients (mean age, 65.3 [range 44–89] years; *p* < 0.001). Family history of cancer was significantly higher in IC compared with OOC patients (*p* < 0.001; Table 1). The majority of patients were diagnosed with Stage I (120/162, 74.7%) and with hormone receptor-positive/HER2-negative (138/167, 82.6%) breast tumors.

**Table 1:** Demographic and clinical characteristics of 192 patients with breast cancer treated at a community hospital who were in- (IC) or out-of-criteria (OOC) with NCCN guidelines for germline testing

	IC (*N* = 104)	OOC (*N* = 88)	*p*
	*N* (%)	*N* (%)	
Patient sex			0.061
Female	98 (94.2)	88 (100.0)	
Male	6 (5.8)	0 (0.0)	
Clinician-reported race/ethnicity			0.306
Asian	3 (2.9)	0 (0.0)	
Black/African American	3 (2.9)	1 (1.1)	
Hispanic or Latino—other	0 (0.0)	1 (1.1)	
Hispanic or Latino—unknown	1 (1.0)	0 (0.0)	
White	98 (94.2)	86 (97.8)	
Family history of cancer (breast, ovary, pancreas or prostate)			< 0.001
Yes	85 (81.7)	46 (52.3)	
No	19 (18.3)	42 (47.7)	
Personal history of nonbreast cancer			0.791
Yes	8 (7.7)	5 (5.7)	
No	96 (92.3)	83 (87.5)	
Stage			0.058
0	10 (9.6)	13 (14.8)	
I	63 (58.7)	59 (67.0)	
II	14 (13.5)	8 (9.1)	
III	6 (5.8)	3 (3.4)	
IV	9 (8.7)	0 (0)	
Unknown	4 (3.7)	5 (5.7)	
Hormone receptor (HR)/*ERBB2* status			0.504
HR+/*ERBB2*−	75 (72.1)	63 (71.6)	
HR+/*ERBB2*+	4 (3.8)	4 (4.5)	
HR−/*ERBB2*+	7 (6.7)	3 (3.4)	
HR−/*ERBB2*−	9 (8.7)	4 (4.5)	
Unknown	9 (8.7)	14 (15.9)	

**Fig. 1:**
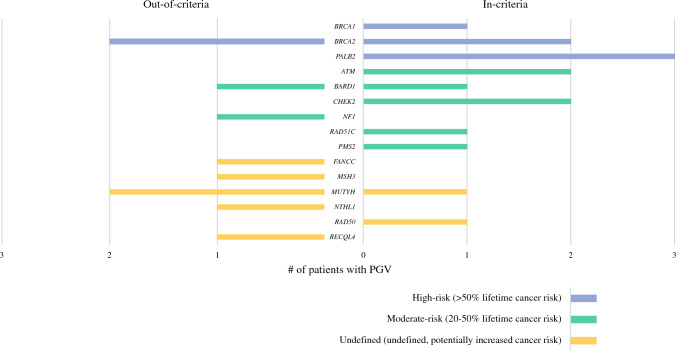
Array of PGV (*N* = 25) in patients who are in- or out-of-criteria. Risk level defined by absolute/lifetime risk for cancer: High (> 50%), Moderate (20–50%), Undefined (undefined, potentially increased). The frequencies of high-and moderate-risk genes were higher in IC (40% and 47%) than OOC patients (20% for each class), although these differences did not reach significance (*p* = 0.70 high-risk, *p* = 0.10 moderate-risk)

Overall, 25 patients (13.0%) had a PGV in one of 15 genes (Fig. 1). An additional 46 (24.0%) patients had ≥ 1 variant(s) of uncertain significance (VUS) in the absence of a PGV finding; the remaining 121 (63%) patients were negative. The frequency of PGVs did not differ significantly between IC (15/104, 14.4%) and OOC patients (10/88, 11.4%, *p* = 0.495). The frequencies of high- (*p* = 0.70) and moderate-penetrance (*p* = 0.10) genes were higher in IC (40% and 47%) than OOC patients (20% for each class). Overall, 16 of 25 (64.0%) patients had PGVs in genes with NCCN clinical management strategies; the frequencies were 12 of 15 (80%) in IC patients and four of ten (40%) in OOC patients.

**Fig. 2:**
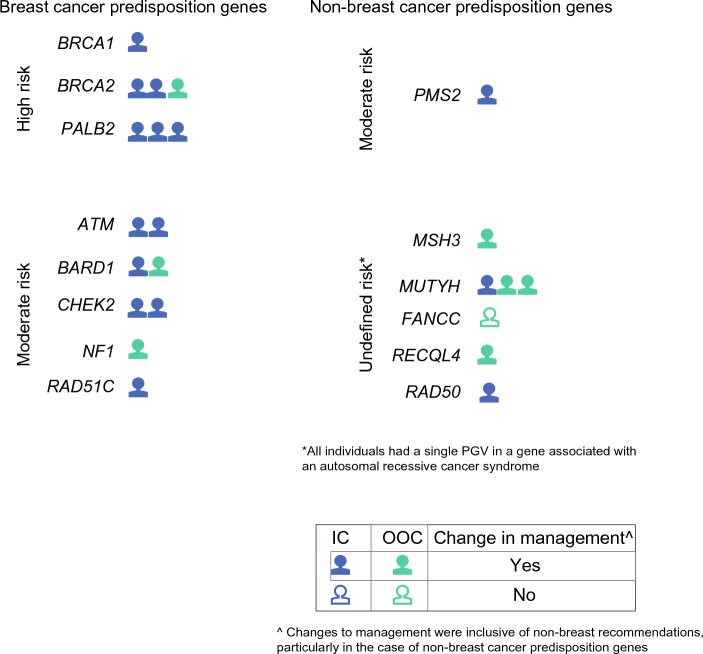
Changes in clinical management in response to GGT. Patients are grouped by gene and breast cancer risk. Two patients with PGV in *BRCA2* and in *NTHL1* had GGT completed after treatment and are not included in this figure. Of the 23 patients shown here, 22 (96.0%) had at least one change to clinical management, primarily related to their breast cancer diagnosis (escalated or de-escalated treatment depending on the breast cancer-related risk of the gene) but also related to screening for other cancers (e.g. colonoscopy for *PMS2* and *MUTYH* carriers).

**Fig. 3:**
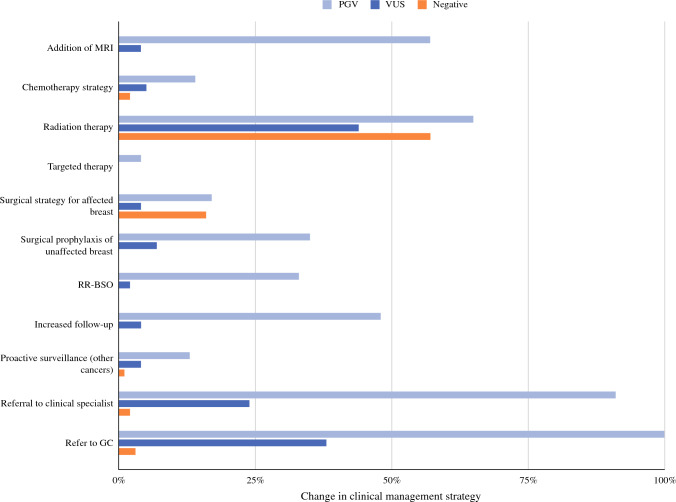
Percentage of patients with changes in clinical management based on return of GGT. Statistically significant differences between patients with PGV compared to those with VUS and negative results were detected for election of BLM, RR-BSO and clinical follow-up (*p* < 0.0001 for all comparisons after multiple testing correction).

Average turnaround time for GGT results was 14 days, resulting in no delay in breast care. Seven patients (3.6%) underwent and received GGT results more than 3 months after their diagnosis and were not included in treatment decision-making analyses. Of the remaining patients, 129 of 185 (69.7%) had at least one GGT-informed change to their clinical management (Fig. 2). The mean number of changes in patients with PGVs (3.0) was significantly higher than that in patients with VUS (0.78) or negative results (0.75, *p* < 0.001). In contrast, the number of management changes in patients with VUS was not significantly different than those with negative results (*p* = 0.81). Additional magnetic resonance imaging, uptake of risk-reducing surgery for the unaffected breast and/or ovaries (risk-reducing bilateral salpingo-oophorectomy [RR-BSO]), and clinical follow-up (increased frequency of visits and/or referral to clinical specialists or genetic counselors) were significantly higher in patients with PGVs compared with those with VUS/negative results (*p* < 0.0001 for all treatments listed; Fig. 3). Only referral to a clinical specialist or genetic counselor was significantly different for patients with VUS compared with negative findings (*p* < 0.0001). Additional genetic counseling for family members was recommended for all PGV-positive patients compared with 17 of 45 (37.8%) patients with VUS and three of 118 (2.5%) patients with negative results.

Among patients who were eligible for unilateral surgical resection, the frequency of BLM varied significantly (*p* = 0.004) between GGT groups (Table 2). Patients with PGVs in *BRCA1/2* were significantly more likely to undergo BLM, whereas those with PGVs in other genes and those with VUS were not significantly more likely to elect for BLM than those with negative results. Of the 16 patients with VUS or negative results who elected for BLM, 12 (75.0%) had one or more of the following risk factors: family history of breast cancer (9/16, 56.3%), young (< 50) age at diagnosis (7/16, 43.8%), and/or additional breast risk factors (multifocal disease, ADH, or dense breasts) (3/16, 18.8%). Twenty (11.8%) patients who were planning BLM or mastectomy chose unilateral mastectomy or BCT after receiving GGT. Patient-reported outcomes indicated that GGT results were useful in shared surgical decision-making for 180 of 185 (97.3%) patients and that knowledge of GGT results before primary therapy reinforced their choice for surgery, especially for 73 of 138 (53%) patients who underwent BCT.

**Table 2:** Odds ratio of overuse bilateral mastectomy and underuse of RT

GGT result	Bilateral mastectomy^a^			Radiation therapy^b^		
	*N* (%)	Odds ratio	95% confidence interval	*N* (%)	Odds ratio	95% confidence interval
Negative	12/103 (11.7)	1 (Reference)		12/78 (15.4)	1 (Reference)	
*BRCA1/BRCA2* PGV	3/3 (100)	51.24	2.5–1051.66	NA	NA	NA
Other PGV	2/15 (13.3)	1.17	0.23–5.81	1/10 (10.0)	0.61	0.07–5.28
VUS	4/35 (11.4)	0.98	0.29–3.26	3/27 (11.1)	0.69	0.18–2.65

Data are numbers with percentages in parentheses unless otherwise indicated

*RT* radiotherapy, *GGT* germline-genetic testing, *PGV* pathogenic germline variants, *VUS* variant of uncertain significance, *NA* not applicable

^a^Eligible cases were patients who had stage 0–III breast cancer

^b^Eligible cases were patients who had stage 0–III breast cancer that underwent breast-conserving therapy, excluding patients > 70 years of age with stage I, estrogen receptor- and/or progesterone receptor-positive/*ERBB2*-negative tumors as defined in Kurian et al.^35^

None of the patients with PGVs in *BRCA1*/*2* underwent BCT. Thus, we could not evaluate compliance with NCCN guidelines for RT in this group. Patients with PGVs in non-BRCA genes or with VUS were not at increased odds for omission of RT compared with those with negative results. For patients who had a change in RT based on GGT, dose escalation (conventional high-dose/standard fractionation and a boost) and de-escalation (APBI, lower dose-accelerated whole-breast irradiation [AWBI] or RT omission) were significantly different between patients with PGVs, VUS, and negative findings (Table 3); 19 of 21 (90.%) and 67 of 67 (100.0%) patients with VUS and negative results, respectively, underwent dose de-escalation compared with four of 15 (26.6%) patients with PGVs.

**Table 3:** Genetics-informed changes in radiation therapy strategy

	Overall *n* = 185	PGV-positive *n* = 25	VUS *n* = 46	Negative *n* = 121	PGV versus VUS/negative, *p*
Total w/change in RT strategy (% of all pts tested)	103 (55.7)	15 (60.0)	21 (45.7)	67 (55.4)	–
APBI or AWBI (%)^a^	81 (76.7)	2 (13.3)	18 (85.7)	61 (91.0)	> 0.001
Omission of RT (%)^a^	9 (8.7)	2 (13.3)	1 (4.8)	6 (9.0)	0.664
Conventional/standard fractionation (%)^a^	13 (12.6)	11 (73.3)	2 (9.5)	0 (0.0)	> 0.001

*APBI* accelerated partial breast irradiation, *AWBI* accelerated whole breast irradiation, *PGV* pathogenic germline variants, *RT* radiotherapy

^a^% of pts with change in RT strategy

## Discussion

In addition to higher BC mortality rates, patients living in rural areas may have the additional challenges of decreased availability, longer travel times to access cancer care, and financial constraints.^36^ GGT as standard-of-care for all patients with BC may reduce disparate survival in rural populations by identifying individuals who may benefit from more intensive treatment approaches, such as BLM, use of precision therapies, or enhanced surveillance to detect early recurrences or second primary cancers. GGT also may identify those patients who can safely undergo treatment de-escalation, including BCT and APBI in those without PGVs in *BRCA1/2*, which may reduce treatment-related side effects, as well as healthcare resource utilization and costs to patients. Potential health economic benefits to patients and the healthcare system warrant further investigation.

To our knowledge, this is the first study to report the impact of incorporating universal GGT as standard-of-care into a rural BC practice. The rate of test uptake (192/210, > 90%) was similar to the 93% reported in a recent cohort of unselected BC patients seen at a comprehensive cancer center.^37^ Of note, in that study, all patients met with a genetic counselor embedded in their multidisciplinary breast clinic, whereas in our study, pre-test education and the return of GGT results was performed by NGEs. A recent study of breast surgeons reported that those without in-house access to genetics services were more likely to use universal GGT.^38^ Moreover, while most respondents were comfortable discussing VUS, fewer were comfortable discussing breast cancer-related PGV and recognized the need for additional genetics education. Mainstreamed genetic testing (MGT) is an approach to GGT that relies on oncology providers, rather than genetic counselors, to provide pre-test education and return of GGT.^39^ The first evaluation of MGT in a breast oncology clinic in the United States found that when used in a large, academic setting, MGT can reduce the number of clinical visits and time to testing and increase test uptake.^40^ The results presented demonstrate that an MGT approach to providing universal GGT can be successfully implemented in a rural setting, despite more limited resources.

The overall (13%), IC (15%), and OOC (11%) prevalence of PGVs in our study from a rural population were similar to those of other universal BC GGT cohorts (overall—average, 12%; range 8–17%; IC average 12%; and OCC average 10%).^20,41–46^ Although PGVs in genes associated with clinical management guidelines were more commonly detected in IC patients in our study, 40% of OOC patients had PGVs in genes for which enhanced surgical, therapeutic, or surveillance interventions are recommended. In addition, 96% of patients with PGVs (100% IC, 88% OOC) had at least one change in clinical management. In a multicenter, prospective study of patients with breast cancer recruited from community and academic centers across the United States, universal GGT impacted both IC and OOC patients, with ≥ 1 clinical management recommendations made for 84% and 68% of IC and OOC patients with a PGV, respectively.^47^ Moreover, clinicians reported that GGT had a positive impact on 37% and 35% of patients with VUS and negative results, respectively, particularly related to recommendation of more conservative surgical strategies. In our study, patients with a VUS had similar clinical-management strategies to those of patients who received negative results, suggesting that receipt of a VUS was not associated with noncompliance with clinical-management guidelines. Similarly, De Silva et al. found that in a cohort of Australian patients with breast cancer, universal GGT resulted in changes in clinical management for 77% of individuals.^48^ Of note, VUS were not reported to patients in this study to avoid anxiety, unnecessary treatments, and undue burden on cancer clinics. Although VUS were reported in our study, nondisclosure of VUS may be an attractive option, especially for nongenetics providers implementing universal GGT.

A number of studies have demonstrated that current guidelines miss patients with actionable PGVs, preventing them from receiving genetics-informed care.^22,37,42,47,48^ It has been suggested that GGT results may result in deviation from management guidelines. In 20,568 women with BC who underwent GGT, Kurian et al. found that women with PGVs in BC predisposition genes were more likely to overuse BLM and chemotherapy and to underuse RT compared with patients with negative results.^35^ While our study found that patients with PGVs in *BRCA1* and *BRCA2* were at increased odds for undergoing BLM compared with those with negative results, patients with PGVs in non-BRCA genes were not significantly more likely to undergo BLM or to omit RT. Thus, universal GGT need not induce guideline-discordant treatment in a rural population. At the same time, GGT enabled RT de-escalation for 42% of BC patients.

Implementation of universal GGT within rural communities may, in fact, improve adherence to treatment guidelines. Multiple studies have found that women living in rural communities were less likely to elect for BCT.^6,49,50^ Whereas distance from RT facilities has been cited as a factor for decreased use of BCT in rural populations, a study of rural patients with BC in Australia found that those who chose mastectomy were more likely to cite fear of recurrence and a belief that a family history of BC was inevitably linked to BC.^49,51–53^ In our study, 12% of patients had a de-escalation of surgery from unilateral radical mastectomy or BLM to BCT based on return of GGT, and the rates of BCT at TOBH increased from 48% before the offering of universal GGT to 79% after universal testing was offered (unpublished data). Thus, the use of GGT in conjunction with shared-decision making may be especially helpful in rural populations to dispel some of the fears or misconceptions about family history associated with guideline-discordant surgery.

This study is limited by small sample size. The 192 patients described in this study represent > 90% of the patients diagnosed with BC at TOBH during a 4-year period; yet the sample size was too small to allow for the analysis of possible underuse of RT in patients with PGVs in *BRCA1* or *BRCA2*. In addition, the recent diagnosis and treatment of this study population (2019–2022) precluded the ability to evaluate whether GGT-informed treatments reduce BC survival disparities that characterize patients living in rural areas. Prospective follow-up of the cohort is warranted. Finally, the patient population serviced by TOBH may not be representative of other rural populations. Many of the counties along the coast of North Carolina are more affluent than other rural areas.^54^ In addition, unlike many rural areas of North Carolina, minority representation in our cohort was low. Patients in other rural regions of North Carolina and across the United States may differ in their willingness to undergo GGT and decisions about clinical management. Future research in additional community practices and rural hospitals is critical to improve our understanding of how ASBrS guidelines-based universal GGT impacts community-based BC care and outcome of patients living in rural areas.

## Conclusions

Universal GGT was successfully implemented in a rural community practice with > 90% test uptake. Return of GGT results (both positive and negative/VUS) changed clinical management for 70% of patients, including changes in surgery, radiation, surveillance, and follow-up. This ability to stratify patients who may benefit from more extensive treatments from those who may be effectively managed by using less aggressive approaches may improve patient outcomes while optimizing medical resource use in rural populations.

### Supplementary Information

Below is the link to the electronic supplementary material.

Supplementary file1 (DOCX 14 KB)
