# Supplementary material for: Universal Germline-Genetic Testing for Breast Cancer: Implementation in a Rural Practice and Impact on Shared Decision-Making
Source: Ann Surg Oncol. 2023 Oct 9;31(1):325–34. doi: 10.1245/s10434-023-14394-3 (PMC10695880; doi:10.1245/s10434-023-14394-3)
Supplement: Supplementary file 1 — Supplementary file1 (DOCX 14 KB) [file 10434_2023_14394_MOESM1_ESM.docx]

# **Table S1. Cancer risk categories for genes with PGV findings**

| **Gene** | **Cancer risk category** | **Supporting information (Pubmed ID or NCCN guideline)** |
| --- | --- | --- |
| *BRCA1* | High | NCCN guidelines for Genetic/Familial High-Risk Assessment: Breast, Ovarian, and Pancreatic, Version 2.2023 |
| *BRCA2* | High | NCCN guidelines for Genetic/Familial High-Risk Assessment: Breast, Ovarian, and Pancreatic, Version 2.2023 |
| *PALB2* | High | NCCN guidelines for Genetic/Familial High-Risk Assessment: Breast, Ovarian, and Pancreatic, Version 2.2023 |
| *ATM* | Moderate | 34254341, 33804961, 33509806  NCCN Guidelines for Genetic/Familial High-Risk Assessment: Breast, Ovarian, and Pancreatic, Version 2.2023 |
| *BARD1* | Moderate | 34424438  NCCN Guidelines for Genetic/Familial High-Risk Assessment: Breast, Ovarian, and Pancreatic, Version 2.2023 |
| *CHEK2* | Moderate | NCCN guidelines for Genetic/Familial High-Risk Assessment: Breast, Ovarian, and Pancreatic, Version 2.2023 |
| *NF1* | Moderate | 23931823, 10762507  NCCN guidelines for Genetic/Familial High-Risk Assessment: Breast, Ovarian, and Pancreatic, Version 2.2023 |
| *PMS2* | Moderate | NCCN guidelines for Genetic/Familial High-Risk Assessment: Colorectal, Version 2.2022 |
| *RAD51C* | Moderate | NCCN guidelines for Genetic/Familial High-Risk Assessment: Breast, Ovarian, and Pancreatic, Version 2.2023 |
| *FANCC* | Undefined | — |
| *MSH3*  *(monoallelic)* | Undefined | — |
| *MUTYH (monoallelic)* | Undefined | — |
| *NTHL1 (monoallelic)* | Undefined | — |
| *RAD50* | Undefined | — |
| *RECQL4 (monoallelic)* | Undefined | — |

High risk, >50% lifetime cancer risk; Moderate risk, 20-50% lifetime cancer risk; Undefined (uncertain, potentially increased cancer risk)
